# *RcRR1*, a *Rosa canina* Type-A Response Regulator Gene, Is Involved in Cytokinin-Modulated Rhizoid Organogenesis

**DOI:** 10.1371/journal.pone.0072914

**Published:** 2013-08-29

**Authors:** Bin Gao, Lusheng Fan, Xingxing Li, Huifang Yang, Fengluan Liu, Ling Wang, Lin Xi, Nan Ma, Liangjun Zhao

**Affiliations:** 1 Department of Ornamental Horticulture and Landscape Architecture, China Agricultural University, Beijing, China; 2 Key Laboratory of Plant Molecular Physiology, Institute of Botany, Chinese Academy of Sciences, Beijing, China; RIKEN Biomass Engineering Program, Japan

## Abstract

*In vitro*, a new protocol of plant regeneration in rose was achieved via protocorm-like bodies (PLBs) induced from the root-like organs named rhizoids that developed from leaf explants. The development of rhizoids is a critical stage for efficient regeneration, which is triggered by exogenous auxin. However, the role of cytokinin in the control of organogenesis in rose is as yet uncharacterized. The aim of this study was to elucidate the molecular mechanism of cytokinin-modulated rhizoid formation in *Rosa canina.* Here, we found that cytokinin is a key regulator in the formation of rhizoids. Treatment with cytokinin reduced callus activity and significantly inhibited rhizoid formation in *Rosa canina*. We further isolated the full-length cDNA of a type-A response regulator gene of cytokinin signaling, *RcRR1*, from which the deduced amino acid sequence contained the conserved DDK motif. Gene expression analysis revealed that *RcRR1* was differentially expressed during rhizoid formation and its expression level was rapidly up-regulated by cytokinin. In addition, the functionality of *RcRR1* was tested in *Arabidopsis*. RcRR1 was found to be localized to the nucleus in *GFP-RcRR1* transgenic plants and overexpression of *RcRR1* resulted in increased primary root length and lateral root density. More importantly, *RcRR1* overexpression transgenic plants also showed reduced sensitivity to cytokinin during root growth; auxin distribution and the expression of auxin efflux carriers *PIN* genes were altered in *RcRR1* overexpression plants. Taken together, these results demonstrate that RcRR1 is a functional type-A response regulator which is involved in cytokinin-regulated rhizoid formation in *Rosa canina*.

## Introduction

Rose (*Rosa* spp.) is one of the most important and widely grown ornamental plants in the world [1]. Previous studies have shown that rose can be efficiently regenerated through organogenesis from various explants, such as leaves, roots, internodes, petioles and immature seeds, which is a prerequisite for plant reproduction and genetic engineering [2], [3], [4]. Increasing evidence has demonstrated that a precise balance of exogenous hormones is required for the organogenesis. We previously established that a certain concentration of auxin is essential for the formation of the root-like organs named rhizoids from leaf explants in *Rosa canina* [5], [6]. However, the role of cytokinin in this process has not been characterized.

Cytokinins are a class of plant hormones that were first identified by their role, in concert with auxin, in the control of cell division in cultured plant cells [7], [8], [9]. While besides their ability to stimulate cell division, cytokinin regulates diverse aspects of plant growth and development, including embryo and seed development [10], [11], shoot initiation [12], leaf senescence [13], vascular differentiation [14] and response to biotic and abiotic stresses [15], [16]. Because of the biological and agricultural importance of cytokinin [17], [18], it is important to understand the basic mechanism by which they regulate such diverse processes in plant cells. In the past decade, substantial progress has been made in characterizing the cytokinin signal transduction pathway in *Arabidopsis*, including the identification of the genes encoding the proteins controlling the key steps in cytokinin biosynthesis and signaling [19], [20], [21].

Cytokinin signal transduction is based on the two-component phosphorelay signal transduction pathway which mainly includes Histidine Kinases (HKs), Histidine Phosphotransfer Proteins (HPs) and Response Regulators (RRs) [22], [23], [24]. In *Arabidopsis*, response regulators (ARRs) fall into four main groups on the basis of their sequence similarities, domain structure and transcriptional response to cytokinin, including type-A ARRs, type-B ARRs, type-C ARRs and *Arabidopsis* pseudo-response regulators (APRRs) [25], [26]. Type-A ARRs have a short C-terminal sequence, but lack the DNA-binding domain named GARP [27], [28]. In contrast to type-A ARRs, type-B ARRs contain a receiver domain and a long C-terminal extension carrying a DNA-binding domain that is responsible for the regulation of transcription of cytokinin-related genes [29]. Type-C ARRs share less sequence similarity to type-A and type-B ARRs; they do not contain DNA-binding domains and are not transcriptionally regulated by cytokinin [30]. The APRRs lack the conserved residues for phosphorylation and are involved in modulating circadian rhythms [31]. However, the response regulators have not been characterized in ornamental plants.

In this study, we found that cytokinin treatment significantly reduced callus activity and rhizoid formation in *Rosa canina*. Furthermore, we identified a type-A response regulator homologous gene, *RcRR1,* and found that its expression was rapidly induced by cytokinin. Importantly, *RcRR1* overexpression *Arabidopsis* plants showed reduced sensitivity to cytokinin and altered auxin distribution and *PIN* genes expression. These findings provide new insights into the mechanism of cytokinin signaling in regulation of organogenesis in *Rosa canina*.

## Results

### Cytokinin Modulates Rhizoid Organogenesis

**Figure 1:**
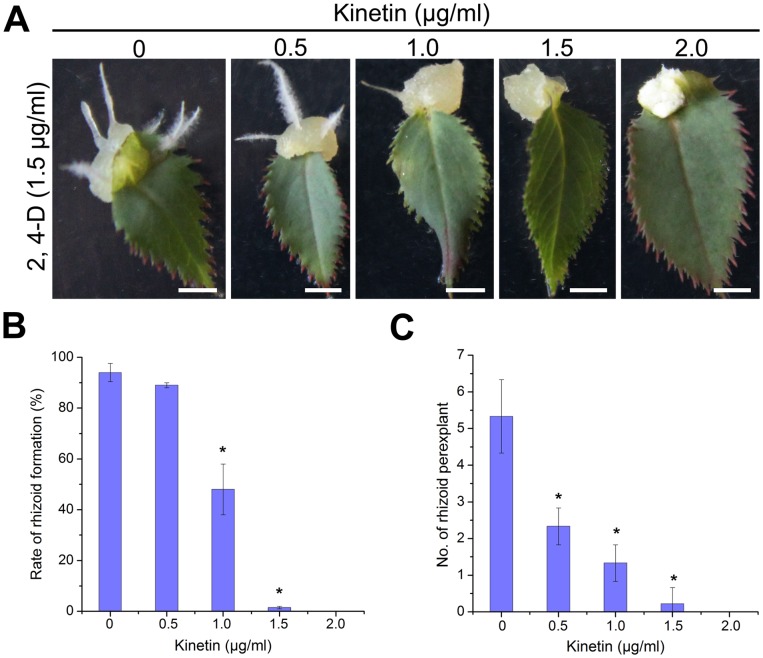
Cytokinin modulates auxin-induced rhizoid organogenesis. (A) Induction of rhizoids by 1.5 µg/ml 2, 4-D at various concentrations of kinetin. Scale bar, 2 mm. (B) Rate of rhizoid formation. (C) Number of rhizoids per explant. Asterisks in (B) and (C) indicate statistically significant differences (Student’s t-test; P<0.01) between the control and cytokinin-treated leaf explants; error bars show SDs.

We have established a new plant regeneration system using protocorm-like bodies (PLBs) that can be induced from the tips of rhizoids and adapted this system to further study the effects of cytokinin on rhizoid formation in *Rosa canina*. As shown in Figure S1, leaf explants of *Rosa canina* were cultivated in Murashige and Skoog (MS) medium containing cytokinin and auxin in various concentrations. The rhizoids could be induced with appropriate auxin concentration even without exogenous cytokinin, while at high levels of auxin, the ability of rhizoid formation decreased (Figure S1). However, we observed a decreased ability of leaf explants to form rhizoids even at low cytokinin levels. With the increasing of cytokinin concentration in the medium, the callus became yellow and gradually disorganized with rare distinguishable root-like structures (Figure 1A and 1B).

Statistical analysis further showed that the number of root-like structures formed per explant decreased 50% at 0.5 µg/ml cytokinin, and the formation of root-like structures was totally inhibited at 2 µg/ml cytokinin (Figure 1C). These results suggest that auxin alone is able to trigger the formation of new organs and that cytokinin plays a regulatory role in organogenesis and ultimately plant regeneration.

### Cloning and Characterization of *RcRR1*

To address the regulatory role of cytokinin in rhizoid formation during *Rosa canina* regeneration, we cloned a type-A ARR homologous gene. A cDNA fragment was amplified using degenerate primers and then the full-length cDNA (accession number KC952001) named *RcRR1* was isolated by rapid amplification of cDNA ends (RACE). The full transcript of *RcRR1* is 939 bp in length and contains a 702 bp open reading frame, a 116 bp 5′-untranslated region (UTR) and a 121 bp 3′-UTR.

**Figure 2:**
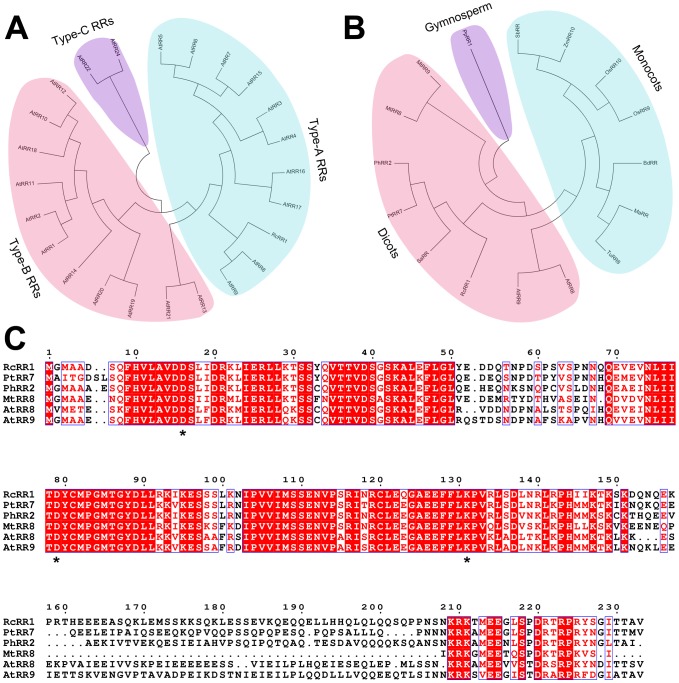
Sequence analysis of the full-length cDNA encoding a *Rosa canina* type-A response regulator, *RcRR1*. (A) Phylogenetic analysis of putative RcRR1 from *Rosa canina* and response regulators in *Arabidopsis*. The aligned sequences were used to construct a phylogenetic tree by MEGA4. (B) Phylogenetic analysis of response regulators from a range of plant species. (C) Sequence alignment of RcRR1 homologs from different species. The alignment was generated using ClustalW and Espript (http://espript.ibcp.fr/ESPript/ESPript/).

To characterize the function of *RcRR1* in *Rosa canina*, we performed phylogenetic analysis using MEGA4. Our analysis showed that *RcRR1* is more closely related to type-A response regulator genes than to other types in *Arabidopsis* (Figure 2A). In addition, RcRR1 was closely related to type-A ARRs from dicots (Figure 2B). Sequence alignment analysis further showed that RcRR1 contains all the features of a type-A response regulator (including the conserved DDK residues and a short C-terminal domain) and shares high similarity with PtRR7 from *Populus trichocarpa* (69% identity), PhRR2 from *Petunia x hybrid* (68% identity), ARR8 from *Arabidopsis* (59% identity), ARR9 from *Arabidopsis* (60% identity) and MtRR8 from *Medicago truncatula* (58% identity) (Figure 2C).

### *RcRR1* Expression is Regulated by Cytokinin

We determined the tissue-specific expression pattern of *RcRR1* by RT-PCR analysis. As shown in Figure S2, the highest level of *RcRR1* expression was seen in roots, suggesting that this gene has a functional importance in roots (Figure S2). Next, we analyzed the expression of *RcRR1* over the course of rhizoid development. Our results showed that there was high expression of *RcRR1* at the early stage of callus formation, and the peak expression level was reached during rhizoid formation, after which the expression of *RcRR1* decreased gradually (Figure S3). These results indicate that *RcRR1* expression has a strong correlation with rhizoid development process.

**Figure 3:**
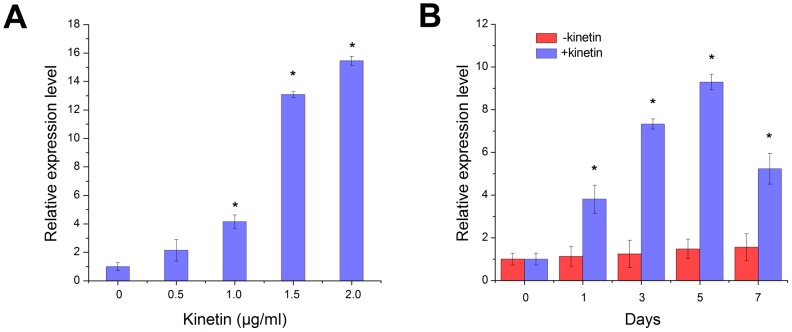
Quantitative real-time PCR shows regulation of *RcRR1* expression by cytokinin. (A) *RcRR1* expression in explants incubated on medium containing 0, 0.5, 1.0, 1.5 and 2.0 µg/ml kinetin for 7 days. (B) Expression of *RcRR1* in response to 0.5 µg/ml kinetin at various incubation times. Asterisks in (A) and (B) indicate statistically significant differences (Student’s t-test; P<0.01) between the control and cytokinin-treated leaf explants; error bars show SDs.

We investigated the effects of exogenous cytokinin treatment on *RcRR1* expression after 1 week of culture on rhizoid formation medium. As the kinetin concentration increased, the level of *RcRR1* expression was significantly up-regulated as compared with the control group (Figure 3A). We then examined *RcRR1* expression in leaf explants on a short time-scale with 0.5 µg/ml kinetin treatment in order to understand how quickly was *RcRR1* response to cytokinin. Our results showed that the expression of *RcRR1* was rapidly up-regulated upon the cytokinin treatment, whereas the expression of *RcRR1* was almost unchanged in the untreated explants (Figure 3B).

We additionally tested the induction of *RcRR1* in callus by a variety of stress treatments. Besides cytokinin, the expression level of *RcRR1* was not affected by any stresses used in this experiment, suggesting that *RcRR1* is specific response to cytokinin (Figure S4). To examine whether cytokinin affects the expression of auxin-related gene, we also homology cloned a *Rosa canina* expressed sequence tag (EST) of the auxin efflux carrier gene from rhizoids and investigated the effect of cytokinin on its expression. RT-PCR showed that the transcription of the putative auxin efflux carrier gene dramatically decreased with increasing cytokinin concentrations (Figure S5).

### RcRR1 Is Localized in the Nucleus

**Figure 4:**
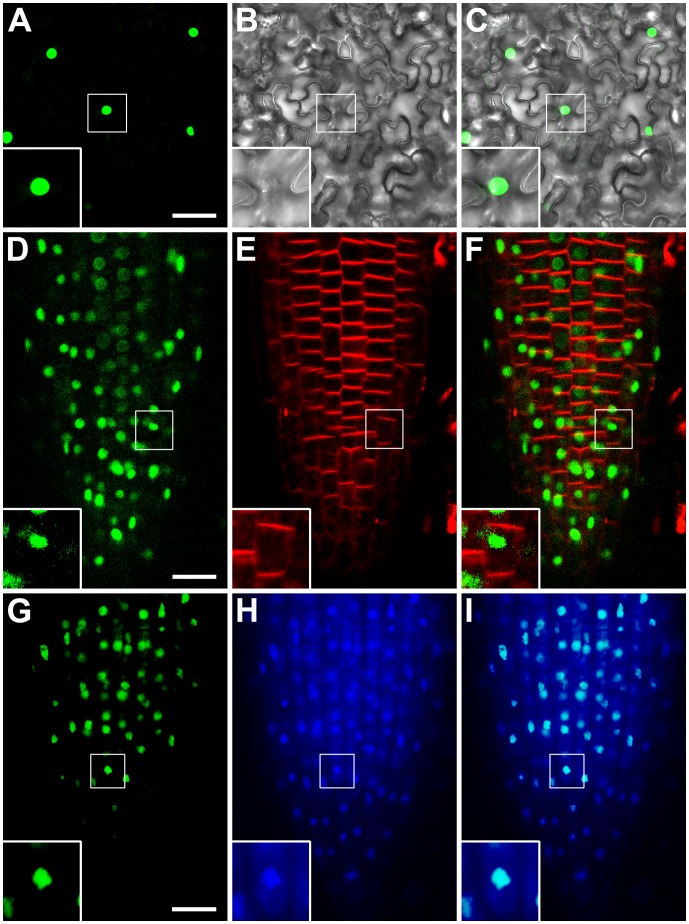
Subcellular localization of *RcRR1*. (A–C) Localization of GFP-RcRR1 in leaf cells of tobacco. (D–I) Localization of GFP-RcRR1 in *Arabidopsis* root cells incubated with FM4-64 (D–F) and DAPI (G–I). Imaging of the 35S::GFP-RcRR1 fusion protein was conducted on a laser scanning confocal microscope. Insets show higher magnification views. Scale bar, 50 µm in (A–C) and 20 µm in (D–I).

To further characterize the function of *RcRR1*, we next investigated the subcellular localization of RcRR1. We used different strategies, including transient expression analysis in *Nicotiana benthamiana* and stable *Arabidopsis* lines transformed with 35S: *GFP*-*RcRR1*. We found that in leaf cells of transiently transformed tobacco, the green fluorescent protein-tagged RcRR1 (GFP-RcRR1) was localized in the nucleus (Figure 4A–C). In transgenic *Arabidopsis,* GFP-RcRR1 also showed a high fluorescent signal as speckles in the nucleus (Figure 4D–F); these results were confirmed through colocalization analysis using the nucleus-specific dye, 4', 6-diamidino-2-phenylindole (DAPI) (Figure 4G–I). All these results suggest that GFP-RcRR1 is a nuclear protein and may act as a transcriptional regulator.

### Characterization of *RcRR1* Overexpression Transgenic *Arabidopsis*

**Figure 5:**
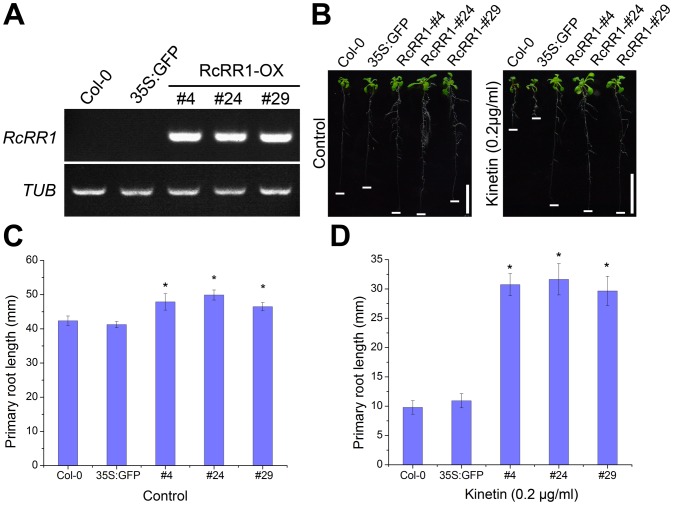
Overexpression of the *RcRR1* gene in *Arabidopsis*. (A) Analysis of *RcRR1* transcripts by RT-PCR in the wild-type and *35S::GFP-RcRR1* transgenic plants, respectively. *TUBULIN (TUB)* was used as an internal control. (B) Phenotypes of 10-day-old seedlings of wild-type and transgenic plants treated with or without 0.2 µg/ml kinetin. Scale bar, 1 cm. (C) Statistical analysis of primary root length of wild-type and transgenic plants. (D) Statistical analysis of primary root length of wild-type and transgenic plants treated with 0.2 µg/ml kinetin. Asterisks in (C) and (D) indicate statistically significant differences (Student’s t-test; P<0.01) between the control and transgenic plants; error bars show SDs.

**Figure 6:**
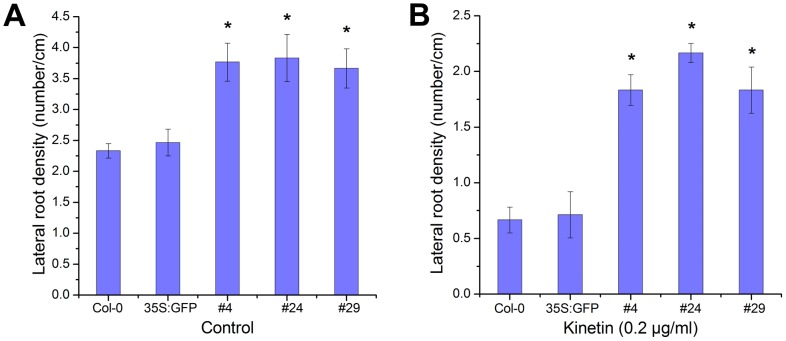
Overexpression of *RcRR1* affects the density of lateral root in transgenic *Arabidopsis*. (A) Statistical analysis of the number of lateral roots (LR) per centimeter of the primary root from the wild-type and transgenic plants. (B) Statistical analysis of the number of LR in the wild-type and transgenic plants treated with 0.2 µg/ml kinetin. Asterisks in (A) and (B) indicate statistically significant differences (Student’s t-test; P<0.01) between the control and transgenic plants; error bars show SDs.

We functionally characterized the role of *RcRR1* in root formation by overexpression of *RcRR1* in *Arabidopsis*. The cDNA fragment containing coding sequences of *RcRR1* was fused in-frame to a GFP sequence, and the *GFP-RcRR1* fusion gene was placed under the control of a cauliflower mosaic virus (CaMV) 35S promoter. All of these transgenic lines showed high expression levels of *GFP-RcRR1* as revealed by RT-PCR (Figure 5A). The *RcRR1-*OX transgenic plants had a longer primary root than that of the wild type (Figure 5B). Further statistical analysis revealed that the transgenic line #24 had the longest primary root (16% longer than that of wild-type plants) (Figure 5C). As shown in Figure 6A, *RcRR1* overexpression also resulted in significantly increased lateral root numbers as compared with wild-type plants. The #24 transgenic plants showed the strongest phenotype with 39% more lateral roots than wild-type plants (Figure 6A).

In addition, we also investigated the sensitivity of *RcRR1-*OX plants to cytokinin. We found that the primary root length, lateral root density and primary root elongation rate of transgenic plants showed varying degrees of reduced sensitivity to cytokinin in comparison with the wild type (Figure 5B; Figure S6 and S7). Treatment with 0.2 µg/ml kinetin caused more than 79% inhibition of primary root length (Figure 5C and 5D) and 71% decrease of lateral root numbers (Figure 6A and 6B) in wild-type plants; however, this dose had a significantly lower effect on the primary root length and lateral root density of *RcRR1-*OX lines. Of these three lines, the #24 transgenic plants showed the strongest phenotype, with primary root length and lateral root density reduced by 40% and 45%, respectively (Figure 5C and 5D; Figure 6A and 6B).

### Auxin Signaling is Altered in the *RcRR1*-OX *Arabidopsis* Root

**Figure 7:**
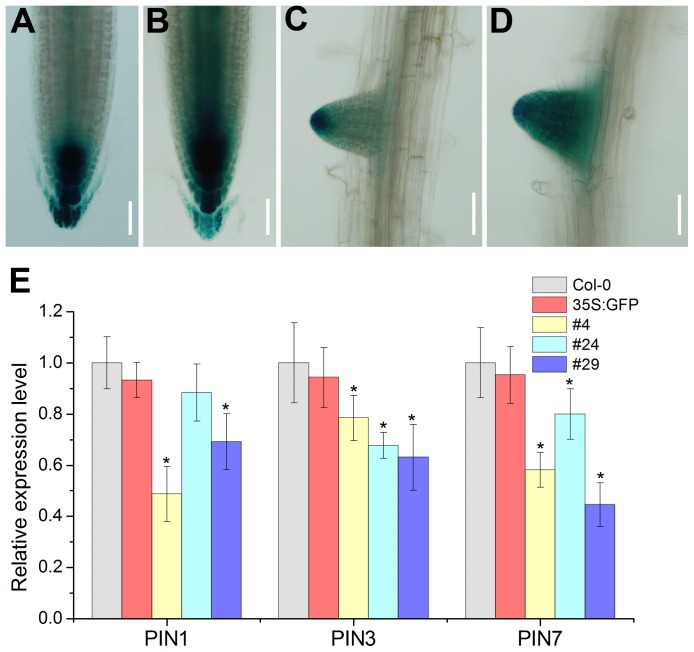
Auxin signaling is altered in the *RcRR1-*OX *Arabidopsis*. (A, B) DR5::GUS expression in primary root tips of 6-day-old seedlings of wild-type (A) and transgenic (B) plants. Scale bar, 50 µm. (C, D) DR5::GUS expression in lateral roots of 6-day-old seedlings of wild-type (C) and transgenic (D) plants. Scale bar, 50 µm. (E) Expression of auxin efflux carrier genes in the wild-type and transgenic plants. *Ubiquitin* (*UBQ*) was used as an internal control.

DR5::GUS is a useful reporter gene, which is widely used for visualizing the distribution of auxin [32], [33]. To investigate whether *RcRR1-*OX affects the distribution of auxin in *Arabidopsis* root, the DR5::GUS reporter was transformed into wild-type plants and hybridized with T2 seedlings of *RcRR1-*OX *Arabidopsis*. In the wild type, DR5::GUS staining was observed in the root cap and quiescent center (Figure 7A). In contrast, DR5::GUS showed a wider distribution in *RcRR1-*OX plants which extended to the pericycle and endodermis (Figure 7B). The broadening distribution of auxin was also observed in the lateral roots of *RcRR1-*OX plants (Figure 7C and 7D). We further studied the effect of *RcRR1-*OX on the expression of the *PIN1, PIN3* and *PIN7* genes. As shown in Figure 7E, the expression of these three genes was significantly down-regulated in the *RcRR1-*OX *Arabidopsis* in comparison with the wild type (Figure 7E). Our results suggest that *RcRR1* might be correlated with the auxin signaling to control root organogenesis.

## Discussion

Rapid and efficient regeneration of rose *in vitro* is very important for rose reproduction. We have reported a new way of plant regeneration via protocorm-like bodies (PLBs), which were induced from rhizoids developed from leaf explants of *Rosa canina* [6]. It is well known that *in vitro* plant regeneration via embryogenesis or organogenesis is dependent on the hormone composition of the culture medium [34]. Although it has been proved that auxin is essential for rhizoid initiation, the role of cytokinin in this process remained unclear. It has been reported that the phytohormone cytokinin modulates multiple plant developmental processes, including cell division and differentiation [35], [36], apical dominance [37], [38] and light responses [39]. In this study, we found that cytokinin treatment alone was unable to trigger any organogenic response but reduced callus activity. Moreover, cytokinin significantly inhibited the auxin-induced rhizoid formation. Yet, we can’t explain the precise mechanism of the link between callus activity and rhizoid output. The most probable explanation is that cytokinin treatment changed hormone balance in callus cells, reduced callus activity and ultimately inhibited rhizoid formation. These findings suggest that cytokinin is a key regulator of rhizoid development.

Type-A ARRs have been shown to participate in various cytokinin-related plant growth and development processes [40], [41]. In *Arabidopsis*, *ARR4* overexpression confers hypersensitivity to red light, indicating a role in light-regulated development [42]. Overexpression of a rice type-A ARR gene also results in hyposensitivity to cytokinin in callus formation [43]. In the present study, we isolated a putative response regulator gene named *RcRR1* and found that the deduced amino acid sequence of RcRR1 showed high similarity to the type-A ARRs in plants and contained the conserved DDK domain indicating that RcRR1 belonged to the type-A ARRs subfamily. Phylogenetic analysis further showed that the response regulator genes were distributed in a wide range of plants including dicots, monocots and gymnosperms, indicating their important function in various species.

In *Arabidopsis*, transcription of most of the type-A ARRs has been detected in all adult organs, and the highest levels are found in the root [44]. In this study, we found that *RcRR1* was also mainly expressed in the root, followed by the stem and leaves. This high expression of *RcRR1* in the root indicates that it may have a function during root-organ formation; moreover, there was a high expression level of *RcRR1* at an early stage of callus formation, which was closely correlated with the rhizoid formation process, suggesting that *RcRR1* is involved in rhizoid organogenesis.

The expression of some type-A ARRs genes is regulated by various environmental stresses, nitrogen levels and phosphate starvation [45], [46], [47]. Our study showed that cytokinin substantially increased the transcript level of *RcRR1*. The up-regulating effect of cytokinin on *RcRR1* expression was observed after short-term treatment, suggesting that *RcRR1* expression can be rapidly regulated by cytokinin, which is consistent with the results in *Arabidopsis*, *M. truncatula,* and *P. trichocarpa.* Additionally, the development of primary and lateral roots of *RcRR1* overexpression transgenic *Arabidopsis* showed reduced sensitivity to cytokinin, further demonstrating that RcRR1 is a functional negative response regulator of cytokinin signaling.

The interaction between auxin and cytokinin in plant organogenesis has been well-established [48], [49], [50]. However, the molecular mechanism of their interaction in controlling plant organogenesis remains uncharacterized. Pernisová *et al*. found that cytokinin modulates auxin-induced root-like organs in *Arabidopsis* via regulation of the auxin efflux [51]. PIN1, PIN3 and PIN7 have been identified as the principal root-specific auxin efﬂux carriers controlling the auxin distribution during root development [33]. We found that the expression pattern of DR5 was dramatically changed in the primary root tip and lateral roots of the *RcRR1-*OX plants. Furthermore, the expression of *PIN1, PIN3* and *PIN7* genes was down-regulated in *RcRR1-*OX *Arabidopsis* as compared with the wild type. In addition, the expression of the putative auxin efflux carrier gene in *Rosa canina* was decreased under cytokinin treatment. Taken together, these results indicate that cytokinin might be associated with the auxin signaling in controlling rhizoid organogenesis via *RcRR1* in *Rosa canina*.

## Materials and Methods

### Plant Materials and Growth Conditions

*Rosa canina* was used in this study. Stem segments from 1-year-old shoots were disinfected according to Tian *et al*. (2008) [6]. Single node segments were cultured on MS media supplemented with 1.0 mg/l 6-benzyladenine (6-BA), 0.004 mg/l a-naphthalene acetic acid (NAA) and 0.1 mg/l gibberellic acid (GA3) at 25°C with a 16 hr light/8 hr dark regimen and a light intensity of 50 mEm^−2^/s. Leaves of 6-week-old plantlets were collected and placed with their adaxial sides down onto rhizoid formation medium as described previously.

### Cytokinin Treatment

To investigate the role of cytokinin in the formation of rhizoids, the leaf explants were cultured on rhizoid formation medium containing 0, 0.5, 1.0, 1.5 and 2.0 µg/ml kinetin. Rhizoid formation was analyzed after 3 weeks of culture.

### Isolation of the Full-length Coding Sequence for *RcRR1*

RNA was extracted from the callus with rhizoids of *Rosa canina* using RN09-EASY spin Kit (Biomed, China) and digested with RNase-free DNaseI. cDNA was synthesized using Superscript III reverse transcriptase (Invitrogen, USA). Primers were designed for *RcRR1* cloning based on the sequences regions of type-A ARRs genes that were conserved among *Arabidopsis*, *P. trichocarpa* and *M. truncatula*. After obtaining a partial fragment using forward primer P_for and reverse primer P_rev, the 3′ and 5′ sequence of *RcRR1* were amplified by the RACE method. The *RcRR1* full-length cDNA was isolated by combining the above three fragments. The products amplified using PrimeStarHS DNA Polymerase (Takara, Japan) were cloned into the pEASY-Blunt Simple Cloning Vector (Trans, China) and verified by sequencing (Sangon Biotech, Shanghai, China). Primers sequences used for the constructs are listed in Table S1. Sequence alignment and phylogenic analysis were performed using the ClustalW, ESPript programs (http://www.genome.jp/tools/clustalw/) and MEGA4 program. Accession numbers used for the phylogenic analysis are listed in Table S2.

### RNA Preparation and Real-Time Quantitative PCR Analysis

For *RcRR1* transcripts analysis, total RNAs were extracted from *Rosa canina* leaf explants treated with various concentrations of kinetin for various lengths of time using the RN09-EASY spin Kit (Biomed, China). The DNaseI-treated RNA (2 µg) of each tissue was reverse transcribed using Superscript III reverse transcriptase (Invitrogen, USA). Real-time Quantitative PCR was performed on an ABI 7500 Real-Time PCR System (Applied Biosystems, USA) using SYBR® Premix Ex Taq™ II (TaKaRa, Japan). The reaction procedure was as follows: denature at 95°C for 30 s, followed by 40 cycles of 5 s at 95°C, 34 s at 58°C. The *Rosa canina 18S rRNA* gene was used as an internal control for normalization, and the data were analyzed by 7500 Software version 2.0.4. The relative expression of the detected genes was calculated using the relative 2^−ΔΔCT^ method [52]. Primers used for qRT-PCR are listed in Table S1.

### Vector Construction and Plant Transformation

The open reading frame (ORF) of *RcRR1* was amplified with primers PstI_for and BamHI_rev. After digestion with *BamH*I and *Pst*I, the fragment was inserted into pCambia 2300-GFP to obtain the p2300-GFP-RcRR1 expression vector for tobacco and *Arabidopsis* transformation. Tobacco leaves were transiently transformed via *A. tumefaciens* leaf infiltration according to Batoko *et al*. (2000) [53]. *Arabidopsis* were infiltrated with *Agrobacterium* using the floral-dip method [54].

The DR5::GUS expression vector was constructed as follows: the DR5 promoter was amplified from the DR5::GFP plasmid and subcloned into pBI121-GUS to produce the DR5::GUS expression vector. Primer sequences used for the constructs are listed in Table S1.

### Cytokinin Response Analysis

For the assay of primary root length and lateral root density, *Arabidopsis* seedlings were grown vertically on 1/2 MS medium supplemented with appropriate concentrations of kinetin. Root length was measured 10 days after germination using Image J software (http://rsb.info.nih.gov/ij/). The lateral root number was counted and converted into the number of lateral roots per centimeter of the primary root.

### Microscopy

For analysis of the subcellular localization of RcRR1, transiently transformed tobacco leaves were cut out, mounted onto glass slides, and viewed under confocal laser scanning microscope (CLSM). Stable transformed GFP-RcRR1 *Arabidopsis* seedlings were incubated with FM4-64 (red) and DAPI (blue) for 3 min and 15 min respectively before CLSM observation.

For analysis of DR5::GUS activity, the 6-day-old seedlings were incubated with GUS staining solution at 37°C for 1 hr and observed under the microscope. Images were processed using Image J software and Adobe Photoshop CS3.

## Supporting Information

Figure S1 **Rhizoids and callus induced by cytokinin and auxin.** Effect of various combinations of 2, 4-D and kinetin on the formation of rhizoids and callus from primary leaf explants. Scale bar, 1 cm.
(TIF)

Figure S2 **Analysis of** ***RcRR1*** **transcript in different tissues**. The expression of *RcRR1* in roots (R), shoots (S), leaves (L), and flowers (F) was analyzed by real-time PCR. *18S rRNA* was used as a control. *18S rRNA* and *RcRR1* genes were amplified for 28 cycles.
(TIF)

Figure S3 **Analysis of** ***RcRR1*** **transcript during rhizoid development**. The expression of *RcRR1* in leaflets (LE), callus-early (CE), callus-mid (CM), callus-late (CL), and rhizoids (RH) was anayzed by real-time PCR. *18S rRNA* was used as a control. *18S rRNA* and *RcRR1* genes were amplified for 28 cycles.
(TIF)

Figure S4 **Expression of the** ***RcRR1*** **gene under various stress conditions.** Callus were subjected to the treatments of 20 µM gibberellin, 200 mM NaCl, 50 µM ABA, low temperature and 9 µM kinetin for 3 hr before collecting samples for RNA preparation. GA3, gibberellin; Na, NaCl; A, ABA; LT, low temperature; KT, kinetin; Un, untreated. *18S rRNA* was used as a control. *18S rRNA* and *RcRR1* genes were amplified for 28 and 30 cycles, respectively.
(TIF)

Figure S5 **Cytokinin modulates the expression of a putative** ***Rosa canina*** **auxin efflux carrier gene.** Expression of putative auxin efflux carrier gene (*RcPIN*) in explants incubated on medium containing 0, 0.5, 1.0, 1.5 and 2.0 µg/ml kinetin for 7 days. *18S rRNA* was used as a control. Primers used for RT-PCR are listed in Table S1. *18S rRNA* and putative *RcPIN* genes were amplified for 28 and 32 cycles, respectively.
(TIF)

Figure S6 **Reduced sensitivity of** ***RcRR1-*** **OX** ***Arabidopsis*** **to cytokinin in primary root growth.** Asterisks indicate statistically significant differences (Student’s t-test; P<0.01) between the control and transgenic plants; error bars show SDs.
(TIF)

Figure S7 **Reduced cytokinin sensitivity of** ***RcRR1-*** **OX** ***Arabidopsis*** **in primary root elongation rate.** Seedlings were grown for 3 days in 1/2 Murashige and Skoog (MS) medium and then transferred to the medium containing 0.2 µg/ml kinetin, and were grown for another 4 days before measurement of primary root length. Asterisks indicate statistically significant differences (Student’s t-test; P<0.01) between the control and transgenic plants; error bars show SDs.
(TIF)

Table S1 **List of primers used in this study.**
(DOCX)

Table S2 **List of accession numbers used in** **Figure 2** **.**
(DOCX)
