# Supplementary figures and images for: RcRR1, a Rosa canina Type-A Response Regulator Gene, Is Involved in Cytokinin-Modulated Rhizoid Organogenesis
Source: PLoS One. 2013 Aug 29;8(8):e72914. doi: 10.1371/journal.pone.0072914 (PMC3757009; doi:10.1371/journal.pone.0072914)

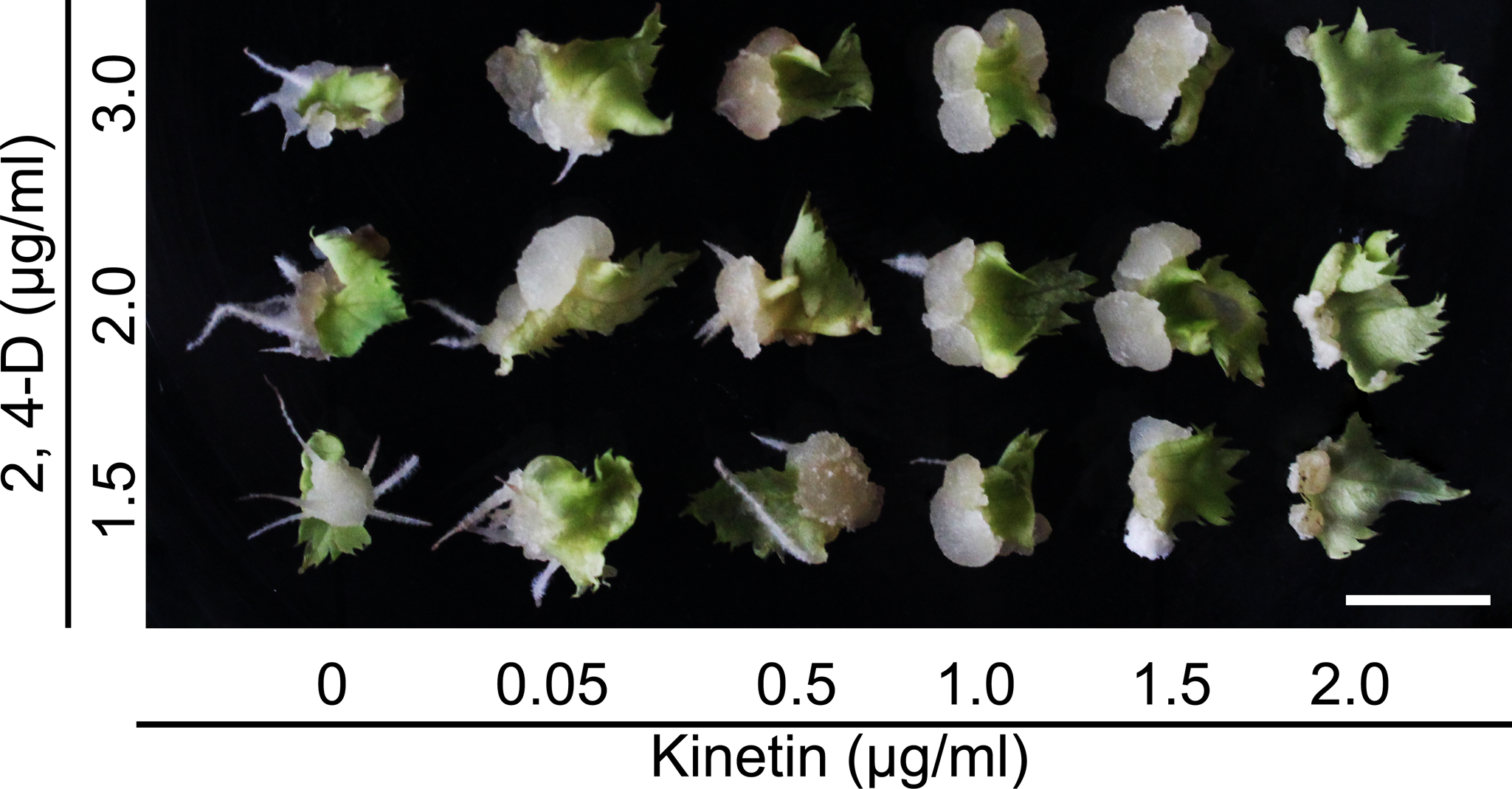

Supplement: Figure S1 — Rhizoids and callus induced by cytokinin and auxin. Effect of various combinations of 2, 4-D and kinetin on the formation of rhizoids and callus from primary leaf explants. Scale bar, 1 cm. (TIF) [file pone.0072914.s001.tif]

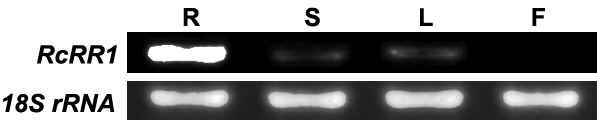

Supplement: Figure S2 — Analysis of RcRR1 transcript in different tissues. The expression of RcRR1 in roots (R), shoots (S), leaves (L), and flowers (F) was analyzed by real-time PCR. 18S rRNA was used as a control. 18S rRNA and RcRR1 genes were amplified for 28 cycles. (TIF) [file pone.0072914.s002.tif]

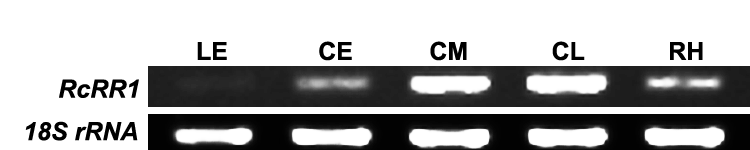

Supplement: Figure S3 — Analysis of RcRR1 transcript during rhizoid development. The expression of RcRR1 in leaflets (LE), callus-early (CE), callus-mid (CM), callus-late (CL), and rhizoids (RH) was anayzed by real-time PCR. 18S rRNA was used as a control. 18S rRNA and RcRR1 genes were amplified for 28 cycles. (TIF) [file pone.0072914.s003.tif]

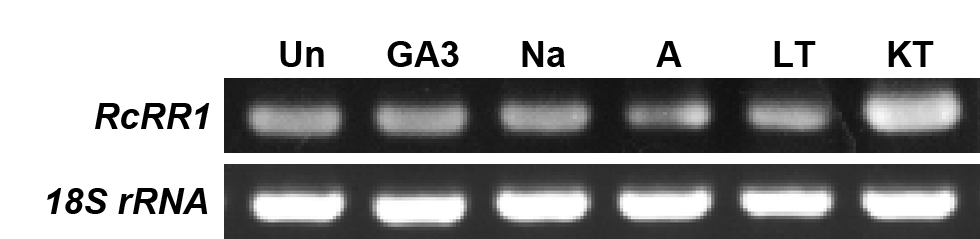

Supplement: Figure S4 — Expression of the RcRR1 gene under various stress conditions. Callus were subjected to the treatments of 20 µM gibberellin, 200 mM NaCl, 50 µM ABA, low temperature and 9 µM kinetin for 3 hr before collecting samples for RNA preparation. GA3, gibberellin; Na, NaCl; A, ABA; LT, low temperature; KT, kinetin; Un, untreated. 18S rRNA was used as a control. 18S rRNA and RcRR1 genes were amplified for 28 and 30 cycles, respectively. (TIF) [file pone.0072914.s004.tif]

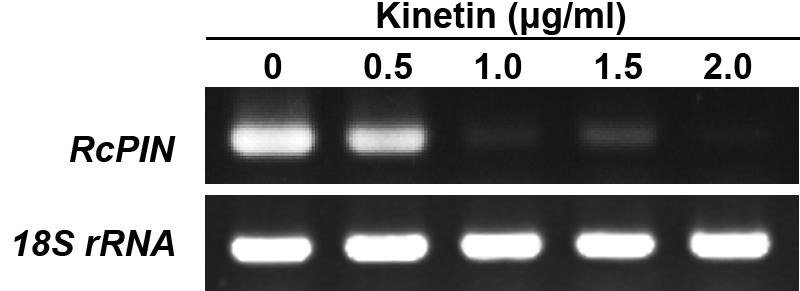

Supplement: Figure S5 — Cytokinin modulates the expression of a putative Rosa canina auxin efflux carrier gene. Expression of putative auxin efflux carrier gene (RcPIN) in explants incubated on medium containing 0, 0.5, 1.0, 1.5 and 2.0 µg/ml kinetin for 7 days. 18S rRNA was used as a control. Primers used for RT-PCR are listed in Table S1. 18S rRNA and putative RcPIN genes were amplified for 28 and 32 cycles, respectively. (TIF) [file pone.0072914.s005.tif]

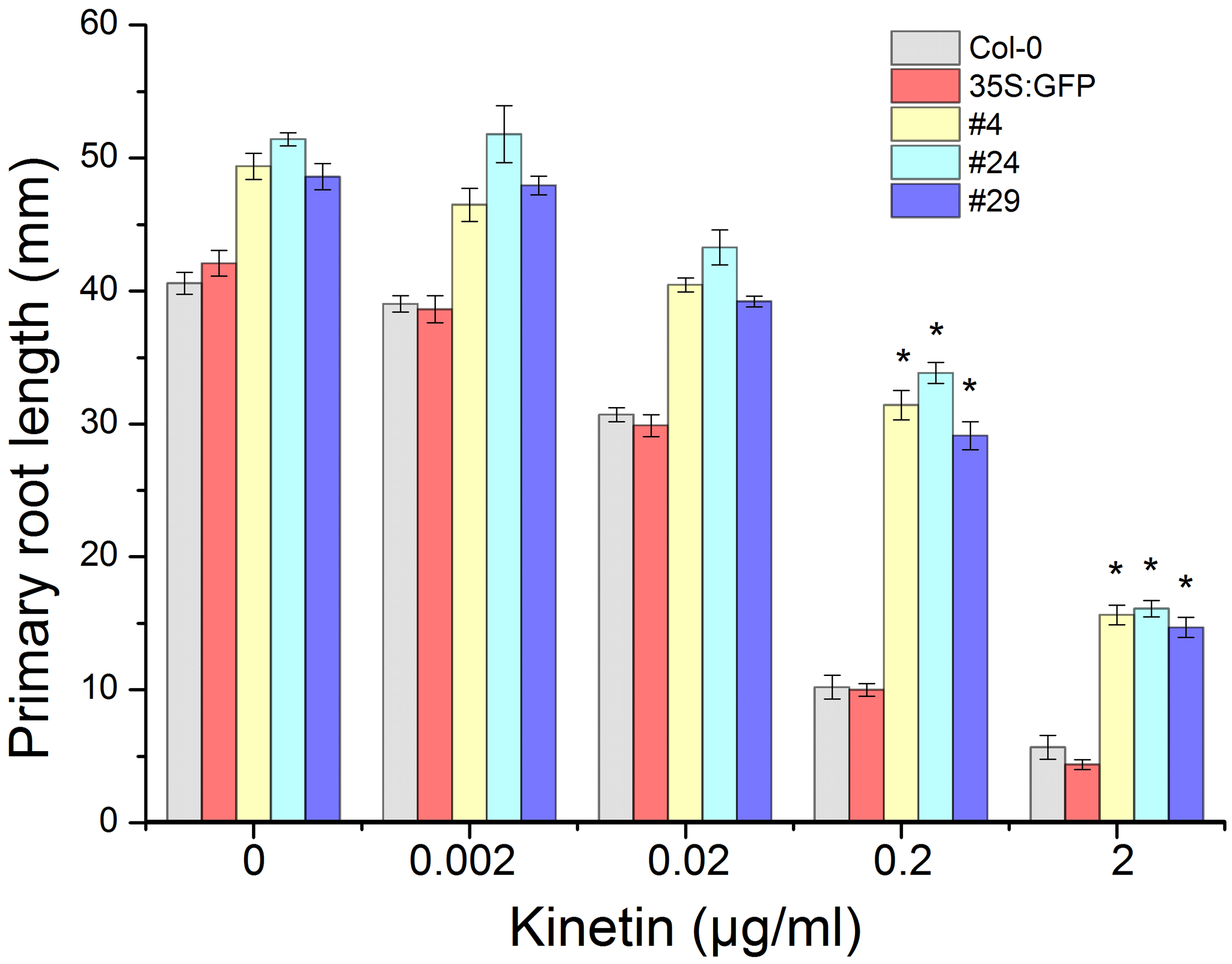

Supplement: Figure S6 — Reduced sensitivity of RcRR1- OX Arabidopsis to cytokinin in primary root growth. Asterisks indicate statistically significant differences (Student’s t-test; P<0.01) between the control and transgenic plants; error bars show SDs. (TIF) [file pone.0072914.s006.tif]

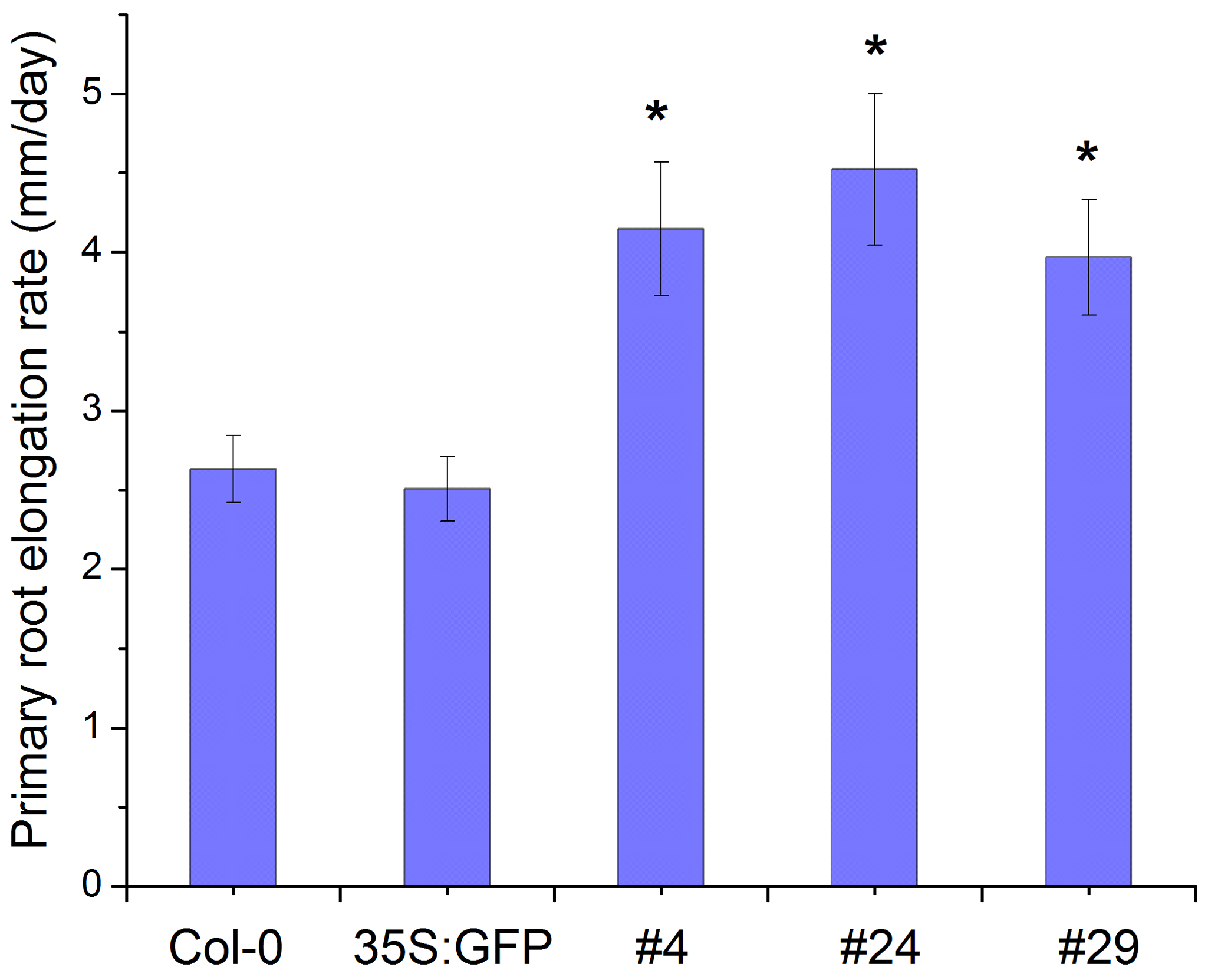

Supplement: Figure S7 — Reduced cytokinin sensitivity of RcRR1- OX Arabidopsis in primary root elongation rate. Seedlings were grown for 3 days in 1/2 Murashige and Skoog (MS) medium and then transferred to the medium containing 0.2 µg/ml kinetin, and were grown for another 4 days before measurement of primary root length. Asterisks indicate statistically significant differences (Student’s t-test; P<0.01) between the control and transgenic plants; error bars show SDs. (TIF) [file pone.0072914.s007.tif]
