# Supplementary material for: RcRR1, a Rosa canina Type-A Response Regulator Gene, Is Involved in Cytokinin-Modulated Rhizoid Organogenesis
Source: PLoS One. 2013 Aug 29;8(8):e72914. doi: 10.1371/journal.pone.0072914 (PMC3757009; doi:10.1371/journal.pone.0072914)
Supplement: Table S1 — List of primers used in this study. (DOCX) [file pone.0072914.s008.docx]

**Table S1. List of primers used in this study.**

| Primers | Sequences (5′-3′) |
| --- | --- |
| P_for | 5'- GATTATTCCATGCCCGGCATGAC-3' |
| P_rev | 5'- TTCTCAGAAGACATAATCAC -3' |
| 3'-race_for | 5'- TATGATTTGCTCAGGAAAATC -3' |
| 3'-race_rev | 5'- CTGATCTAGAAGGTACCGGATCC -3' |
| 5'-race_for | 5'- AGATTTGGGHTGAAATYYGGTCRGATCTTGCTTT-3' |
| 5'-race_rev | 5'- AACACCTATTTATTCTTGAAGGCACAT -3' |
| RT_for | 5'-ATGGGGATGGCCGCAGATTCTCAG-3' |
| RT_rev | 5'-TCAAACGGCAGTGGTGATGCCACT-3' |
| RcPIN_for | 5'-TTTATTCATGGCTTTACAACCAAAG-3' |
| RcPIN_rev | 5'-GAGGCAAACACAAAAGGAACAATTC-3' |
| 18S_for | 5'-CGCTACACTGATGTATTCAACGAGC-3' |
| 18S_rev | 5'-ACAATAATCCTTCCGCAGGTTCACC-3' |
| RcRR1 _for | 5'- TTTTGTGGGATGTGGATATTGG -3' |
| RcRR1_rev | 5'- CAGGCGGGTATCAACCAGCAGT -3' |
| BamHI _for | 5'-GTAGGATCCATGGGGATGGCCGCAGATTCTCAG-3' |
| PstI _rev | 5'-CGCCTGCAGTCAAACGGCAGTGGTGATGCCACT-3' |
| TUB_for | 5′-CGTGGATCAAGCAATACAGAGCC-3′ |
| TUB_rev | 5′-CCTCCTGCACTTCCACTTCGTCTTC-3′ |
| PIN1 _for | 5'-TACTCCGAGACCTTCCAACTACG-3' |
| PIN1_rev | 5'-TCCACCGCCACCACTTCC-3' |
| PIN3 _for | 5'-GAGGGAGAAGGAAGAAAGGGAAC-3' |
| PIN3_rev | 5'-CTTGGCTTGTAATGTTGGCATCAG-3' |
| PIN7_for | 5'-CGGCTGATATTGATAATGGTGTGG-3' |
| PIN7_rev | 5'-GCAATGCAGCTTGAACAATGG-3' |
| UBQ10_for | 5'-CACACTCCACTTGGTCTTGCGT-3' |
| UBQ10_rev | 5'-TGGTCTTTCCGGTGAGAGTCTTCA-3' |
