# Supplementary material for: RcRR1, a Rosa canina Type-A Response Regulator Gene, Is Involved in Cytokinin-Modulated Rhizoid Organogenesis
Source: PLoS One. 2013 Aug 29;8(8):e72914. doi: 10.1371/journal.pone.0072914 (PMC3757009; doi:10.1371/journal.pone.0072914)
Supplement: Table S2 — List of accession numbers used in Figure 2 . (DOCX) [file pone.0072914.s009.docx]

**Table S2. List of accession numbers used in Figure 2.**

| Gene Name /Species | accession numbers | Gene Name /Species | accession numbers |
| --- | --- | --- | --- |
| *ARR1/Arabidopsis thaliana* | AEE75876 | *ARR19/Arabidopsis thaliana* | AEE32402 |
| *ARR2/Arabidopsis thaliana* | ABF47279 | *ARR20/Arabidopsis thaliana* | AEE80378 |
| *ARR3/Arabidopsis thaliana* | NP_176202 | *ARR21/Arabidopsis thaliana* | AED91122 |
| *ARR4/Arabidopsis thaliana* | NP_172517 | *ARR22/Arabidopsis thaliana* | AEE74061 |
| *ARR5/Arabidopsis thaliana* | AEE78368 | *ARR24/Arabidopsis thaliana* | AED93557 |
| *ARR6/Arabidopsis thaliana* | NP_201097 | *MtRR9/Medicago truncatula* | XP_003598557 |
| *ARR7/Arabidopsis thaliana* | NP_173339 | *MtRR8/Medicago truncatula* | XP_003627814 |
| *ARR8/Arabidopsis thaliana* | NP_181663 | *PhRR/Petunia x hybrida* | BAL43557 |
| *ARR9/Arabidopsis thaliana* | NP_191263 | *PtRR/Populus trichocarpa* | XP_002322625 |
| *ARR10/Arabidopsis thaliana* | NP_194920 | *SaRR/Striga asiatica* | ABG35780 |
| *ARR11/Arabidopsis thaliana* | AEE34686 | *TuRR8/Triticum urartu* | EMS50922 |
| *ARR12/Arabidopsis thaliana* | NP_180090 | *MaRR6/Musa acuminata* | ABG33766 |
| *ARR13/Arabidopsis thaliana* | AEC07932 | *BdRR/Brachypodium distachyon* | XP_003578944 |
| *ARR14/Arabidopsis thaliana* | AEC05495 | *OsRR9/Oryza sativa* | NP_001065722 |
| *ARR15/Arabidopsis thaliana* | AEE35644 | *OsRR10/Oryza sativa* | NP_001066118 |
| *ARR16/Arabidopsis thaliana* | AEC09862 | *ZmRR10/Zea mays* | ACG32848 |
| *ARR17/Arabidopsis thaliana* | AEE79515 | *SbRR/Sorghum bicolor* | XP_002442679 |
| *ARR18/Arabidopsis thaliana* | AED96995 | *PpRR/Pinus pinaster* | AFU91979 |
